# Band Tailoring Enabled Perovskite Devices for X‐Ray to Near‐Infrared Photodetection

**DOI:** 10.1002/advs.202414259

**Published:** 2025-01-14

**Authors:** Yi‐Chu He, Guan‐Hua Dun, Jun Deng, Jia‐Li Peng, Ken Qin, Jia‐He Zhang, Xiang‐Shun Geng, Min‐Shu Zhang, Ze‐Shu Wang, Yan Xie, Zhao‐Qiang Bai, Dan Xie, He Tian, Yi Yang, Tian‐Ling Ren

**Affiliations:** ^1^ School of Integrated Circuits and Beijing National Research Center for Information Science and Technology (BNRist) Tsinghua University Beijing 100084 China; ^2^ Beijing Superstring Academy of Memory Technology Beijing 102600 China; ^3^ Department of Engineering Physics Tsinghua University Beijing 100084 China

**Keywords:** band tailoring, heterojunction, Te/FAPbI_3_ photodetector, wide‐ spectrum, X‐ray to Infrared

## Abstract

Perovskite semiconductors have shown significant promise for photodetection due to their low effective carrier masses and long carrier lifetimes. However, achieving balanced detection across a broad spectrum—from X‐rays to infrared—within a single perovskite photodetector presents challenges. These challenges stem from conflicting requirements for different wavelength ranges, such as the narrow bandgap needed for infrared detection and the low dark current necessary for X‐ray sensitivity. To address this, this study have designed a type‐II FAPbI_3_ perovskite‐based heterojunction featuring a large energy band offset utilizing narrow bandgap tellurium (Te) semiconductor. This innovative design broadens the detection range into the infrared while simultaneously reducing dark current noise. As‐designed device allows for the detection of near infrared band, achieving a detectivity of 6.8 × 10^9^ Jones at 1550 nm. The low dark current enables X‐ray sensitivity of up to 1885.1 µC Gy⁻¹ cm⁻^2^. First‐principles calculations confirm the type‐II band structure alignment of the heterojunction, and a self‐driven response behavior is realized. Moreover, this study have developed a scalable 40 × 1 sensor array, demonstrating the potential for wide‐spectrum imaging applications. This work is expected to advance the application of perovskite‐based wide‐spectrum devices.

## Graphical abstract

Perovskite‐based photodetectors are improved for wide‐spectrum detection X‐rays to near‐infrared using a type‐II heterojunction with tellurium. This design reduces dark current noise and enhances sensitivity, with a scalable sensor array showing promise for imaging applications.

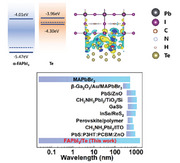

## 1 Introduction

Wide‐spectrum sensing technologies offer significant promise for various applications, including medical diagnostics, security, and environmental monitoring.^[^ ^1^ ^]^ By capturing information across a wide spectrum, such as from X‐rays to near‐infrared, these technologies enable the collection of richer data, thereby enhancing comprehensive sensing capabilities for detailed and accurate assessments.^[^ ^2^ ^]^ Current wide‐spectrum sensing systems are typically composed of discrete sensors, each tailored to capture information within specific wavelength ranges (e.g., CdTe for <0.01 nm at X‐ray,^[^ ^3^ ^]^ Si for 0.4–0.7 nm at visible,^[^ ^4^ ^]^ InGaAs for 1.5–1.7 µm at short‐wave infrared).^[^ ^5^ ^]^ This fragmented data collection process necessitates complex backend algorithms for effective information integration.^[^ ^6^ ^]^ Integrating wide‐spectrum sensing capabilities into a single device offers a prospective solution to these challenges.

Perovskite semiconductors, with their high light absorption coefficients, low effective carrier masses, and long carrier lifetimes, show great potential in the field of photodetection.^[^ ^7^ ^]^ In recent years, various types of perovskites have been utilized for separate X‐ray and visible light detection,^[^ ^8^ ^]^ demonstrating sensitivity beyond silicon counterparts. To further broaden the response spectrum, various efforts such as Pb/Sn alloy engineering, energy band tunning, doping have been employed to enhance long wavelength region absorption.^[^ ^9^ ^]^ However, realizing the X‐ray to near‐infrared wavelength photodetection in a single perovskite device remains challenging due to the different material property demands for photo detection in different wavelength.^[^ ^10^ ^]^ Element doping can lower the bandgap and extend infrared response, but the high‐density defects introduced during doping process often led to carrier scattering and increased dark current, weakening X‐ray detection capability.^[^ ^11^ ^]^ Moreover, perovskite devices using Pb/Sn alloy engineering often face stability issues from Sn oxidation and typically have a relatively large bandgap, which make it difficult to detect infrared light, such as the C‐band at 1550 nm.^[^ ^12^ ^]^

In this work, we demonstrate a sensor capable of detecting from X‐ray to near‐infrared (NIR) on a single device using a Te transmission layer design, which simultaneously reduces dark current noise and broadens the long‐wavelength response spectrum. First‐principles calculations were used to select tellurium (Te) as a charge‐blocking layer in the perovskite, achieving a significant offset that effectively reduces the device dark current level and suppressed the noise current power. The low‐dark current noise facilities an X‐ray sensitivity reaching 1885.1 µC Gy^−1^ cm^−2^. The charge‐blocking layer also possesses a narrow bandgap, enabling the detection of long‐wavelength radiation, with a detectivity at 1550 nm reaching 6.8 × 10^9^ Jones. The band interface features a type‐II heterojunction structure, which facilitates efficient detection under self‐driven conditions. Additionally, a 40 × 1 scale sensor array is fabricated to verify its scalability, and a multi‐wavelength wide‐spectrum imaging application is demonstrated. This work is expected to advance the application of wide‐spectrum devices.

## 2 Results and Discussion

**Figure 1:**
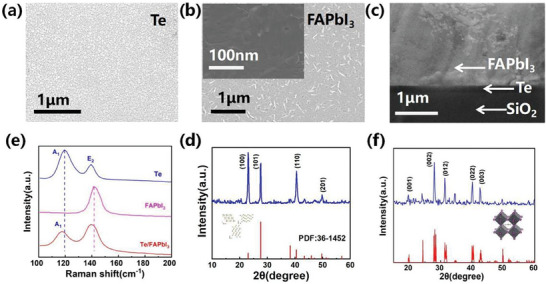
SEM top‐view image of a) tellurium film and b) FAPbI_3_ perovskite film. c) cross‐sectional SEM image of Te/FAPbI_3_ heterojunction. d) Raman spectrum of original Te film, FAPbI_3_ perovskite film and Te/FAPbI_3_ heterojunction. X‐ray diffraction patterns of e) tellurium film^[^ ^18a^ ^]^ and f) α‐FAPbI_3_ perovskite film with scan range of 10–60°. The inset images show the cell structures of tellurium film and α‐FAPbI_3_ respectively.^[^ ^16b^ ^]^

**Figure** **1**a presents the scanning electron microscope (SEM) micrograph of tellurium (Te) film fabricated via thermal evaporation,^[^ ^13^ ^]^ exhibiting compact crystalline morphology. Atomic force microscope (AFM) was employed to examine the Te film surface at high resolution, revealing a root mean square (RMS) roughness of 1.3 nm (Figure S2, Supporting Information).^[^ ^14^ ^]^ The SEM image of FAPbI_3_ film on Te film (Figure 1b) displays a pinhole‐free morphology, which suggests the high quality of perovskite film. Energy dispersive spectrometer (EDS) mapping analysis is conducted to further study the uniformity of FAPbI_3_ film (Figure S3, Supporting Information).^[^ ^15^ ^]^ The well‐distributed Pb and I elements, coupled with the absence of Te signal, confirms the uniform coverage of the perovskite film on Te film. Cross view SEM image in Figure 1c shows a compact contact interface between Te and perovskite film, which will facilitate effective charge transport. The Raman peaks of the individual Te and perovskite components are consistent with those of the heterojunction (Figure 1d). A distinct peak at 142 cm^−1^ in the perovskite film confirms the presence of the black phase of FAPbI_3_.^[^ ^16^ ^]^ The Raman peak of 119 cm^−1^ Te A_1_ mode of individual film shows a slight blue shift compared with heterojunction sample, which can be attributed to the enhanced intra‐chain interactions within the individual Te film.^[^ ^17^ ^]^ In addition, X‐ray diffraction (XRD) analysis (Figure 1e) confirms that the Te film crystallizes in a hexagonal structure (space group: P3_1_21), in agreement with literature results.^[^ ^18^ ^]^ Figure 1f shows the XRD pattern of α‐FAPbI_3_ perovskite film, which is consistent with previously reported results,^[^ ^16b^ ^]^ confirming the trigonal crystal system (space group: P3m1). The inset images illustrate the unit cell structures of Te and α‐FAPbI_3_, respectively. Both XRD patterns confirm the high purity and crystallinity of the Te and perovskite films.

**Figure 2:**
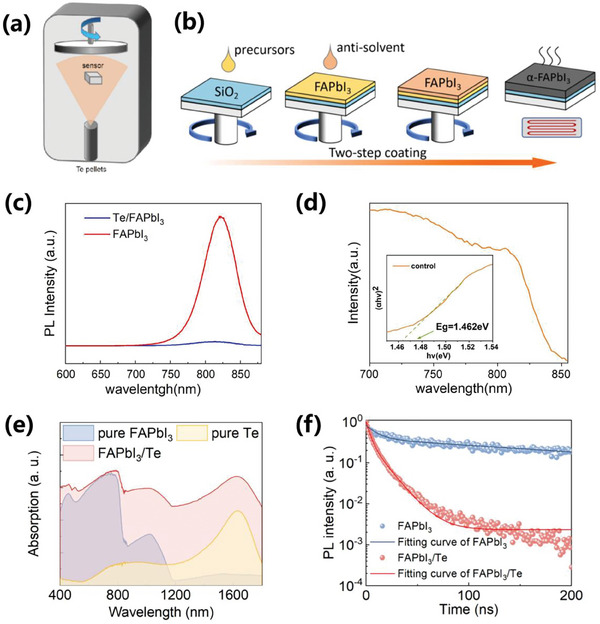
a) Schematic diagram of black‐phase FAPbI_3_ perovskite film prepared by two‐step coating and b) tellurium film deposited by thermal evaporation. c) Photoluminescence spectrum of FAPbI_3_ perovskite film and Te/FAPbI_3_ heterojunction. d) UV‐visible absorption spectrum Inset: Tauc plot. e) UV–vis–IR absorption spectrums of pure FAPbI_3_, pure Te and Te/FAPbI_3_ in the wavelength range of 400–1700 nm. f) The time‐resolved PL of pristine FAPbI_3_ perovskite film (τ_average_ = 241.8 ns) and Te/FAPbI_3_ heterojunction (τ_average_ = 10.8 ns).

**Figure** **2**a,b illustrates the fabrication process of the heterojunction device,^[^ ^19^ ^]^ with detailed procedural steps provided in the Experimental section. Figure 2c depicts Photoluminescence (PL) spectrum of FAPbI_3_ perovskite film and Te/FAPbI_3_ heterojunction.^[^ ^20^ ^]^ Compared with FAPbI_3_ perovskite, the steady PL intensity of Te/FAPbI_3_ heterojunction decreases drastically by ≈96%, which is attributed to sharp photoluminescence quenching due to charge transfer between Te and FAPbI_3_.^[^ ^21^ ^]^ To obtain the bandgap of FAPbI_3_, the data of ultraviolet‐visible‐near‐infrared (UV‐Vis‐NIR) absorption spectrum is analyzed based on Tauc plot method, as shown in Figure 2d.^[^ ^22^ ^]^ Accordingly, the optical band gap is calculated as 1.46 eV, providing theoretical guidance for broadband light response of following heterojunction device. Besides, UV‐Vis‐NIR absorption curve of pure FAPbI_3_, pure Te, and Te/FAPbI_3_ are compared in Figure 2e. The pure perovskite exhibits significant absorption in the 400–900 nm range, with an absorption edge ≈900 nm. This can be attributed to the absorption due to the transition from the valence band to the conduction band in the FAPbI_3_ perovskite.^[^ ^23^ ^]^ The pure Te shows noticeable absorption in the infrared region, from ≈800 nm to 1700 nm, which is consistent with narrower bandgap property of Te (0.34 eV).^[^ ^24^ ^]^ Compared to the pure FAPbI_3_ perovskite material and pure Te material, the heterojunction material demonstrates overall stronger absorption across the UV–vis–NIR (400‐1700 nm), highlighting the effectiveness of the heterojunction structure in achieving broad‐spectrum absorption. In addition, the time‐resolved photoluminescence (TRPL) was utilized to evaluate charge recombination properties (Figure 2f). Specifically, the photoluminescence lifetime decay curve was fitted using a bi‐exponential model, with detailed fitting procedures provided in the Experimental section of the Supporting Information (**Table** 1). The parameters τ_1_/A_1_ and τ_2_/A_2_ represent the fast and slow decay lifetime components and the corresponding proportions, respectively.^[^ ^25^ ^]^ The fast and slow decay lifetimes originate from charge carrier extraction within the Te/perovskite interface, and non‐radiative recombination in perovskite, respectively.^[^ ^26^ ^]^ The fast decay lifetime of Te/perovskite heterojunction (3.7 ns) is shorter than that of pure perovskite sample (10.8 ns), suggesting the effective charge carrier extraction at the Te/perovskite interface.^[^ ^23^, ^27^ ^]^

**Figure 3:**
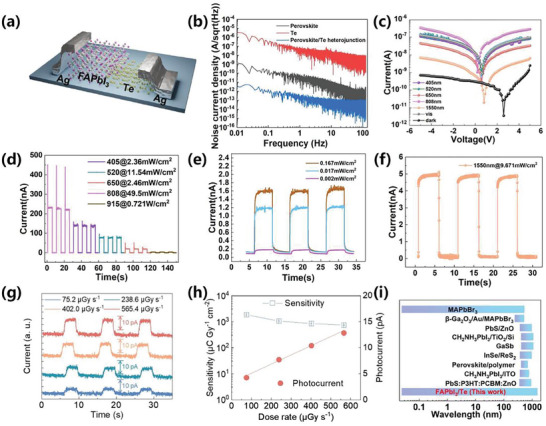
a) Schematic structure of Te/FAPbI_3_ heterojunction photodetector. b) noise power spectrum of perovskite, tellurium and Te/FAPbI_3_ heterojunction photodetector. c) *I*–*V* curves of Te/FAPbI_3_ device under variable illumination wavelengths, including 405, 520, 605, 808, 915, and 1550 nm (Bias: 5 V). d) dynamic photoresponse curves of Te/FAPbI_3_ heterojunction photodetector under periodic illumination with wavelength range from 405 to 915 nm at 5V bias. e) broadband spectrum photoswitching curves of Te/FAPbI_3_ device at a bias of 5V under 808nm laser excitation with different illumination intensities. f) *I*–*T* curve of Te/FAPbI_3_ heterojunction photodetector under X‐ray illumination with dose rate of 75.2 µGy·s^−1,^ 238.6 µGy·s^−1^, 402.0 µGy·s^−1^ and 565.4 µGy·s^−1^. g) Sensitivity and photocurrent of heterojunction device under X‐ray illumination range from 75.2 µGy·s^−1^ to 565.4 µGy·s^−1^. i) benchmark of wavelength detection range of broadband spectrum photodetector.

**Figure** **3**a demonstrates Te/FAPbI_3_ heterojunction device schematically. Figure 3b shows that the heterojunction device exhibits lower noise current density in the frequency range of 0.01–100 Hz compared to the pure perovskite and pure Te devices. This phenomenon can be explained by the fact that a larger energy offset significantly reduces the total noise level by lowering shot noise level.^[^ ^28^ ^]^ Shot noise arises from quantum mechanical fluctuations of charge carriers as they pass through the barrier. An increase in the barrier height makes it more difficult for charge carriers to cross the barrier, thereby reducing the shot noise level.^[^ ^29^ ^]^ Figure 3c,d present current‐voltage (*I*–*V*) curves and current‐time (*I*–*T*) curves of heterojunction photodetector under darkness, 405, 520, 605, 808, 915, and 1550 nm laser illumination, respectively, indicating broad‐spectrum response behaviors. The dark current minimum of 1.2 pA deviates from zero bias, suggesting the formation of a built‐in electric field between the perovskite and Te layers. Figure 3e depicts dynamic *I_on_/I_off_* switching photoresponse of heterojunction device under variable light intensities at a bias of 5 V (808 nm) in a regular ten‐second cycle. With the increase of illumination power, the photoresponse of the device is gradually enhanced. By comparing their performance before and after 5 months of storage in ambient conditions, the long‐term stability of the devices is investigated. Compared to the measurements in fresh states, the devices after 5 months storage retained ≈95% of their original photocurrent response level. In addition, after 5 months of storage, the devices maintained stable photoresponse under 50 cycles of *I_on_/I_off_* illumination measurement.^[^ ^30^ ^]^ The results have proved that the device possesses high stability and repeatability. Furthermore, the time‐resolved response behaviors, including ON/OFF current ratio, the rise and decay time is a crucial indicator to measure a detector. Meanwhile, the detector possesses the capability of detecting near‐infrared (NIR) light showing in the Figure 3f. (Figure S9, Supporting Information) exhibits one periodic illumination photoresponse of Te/FAPbI_3_ photodetector under ON/OFF switch irradiation with 5 V bias to wavelength of 405 nm. Rise time is defined as the interval for the response curve from 10% to 90% of steady‐state value, while decay time is defined as the interval for the response curve from 90% to 10% of steady‐state value.^[^ ^31^ ^]^ Thus, the rise and decay time are determined to be 10.00 and 25.66 ms respectively, which implies the ultrafast response speed. Correspondingly, the *I_on_*/*I_off_* is meanwhile assessed to be on the order of 10^3^. To investigate the performance of the photodetector further, responsivity (*R*) and detectivity (D*) of the heterojunction device are evaluated. The *R* is assigned to represent the ability of a device to convert an optical signal into an electrical signal under incident illumination, which can be derived from the following equation,^[^ ^32^ ^]^

(1) $$R=\frac{I_{p}-I_{d}}{P\cdot A}$$

Among them, *I_p_,I_d_*, *P* and *A* represent photocurrent, dark current, light intensity and active area, respectively. Furthermore, D* describes the ability of detecting signals in a noise back‐ground.^[^ ^33^ ^]^

(2) $$D^{*}=\frac{R}{{\left(2qI_{d}/A\right)}^{1/2}}$$

where *q* is elementary charge and *A* is device area. *I_d_* and *R* are darkcurrent and responsivity, respectively. The high detectivity of 6.8 × 10^9^ Jones confirms a good detection capacity at 1550 nm in NIR region. Additionally, the X‐ray detection performance is measured, and the detailed experiment configuration is described in Experiment section.^[^ ^34^ ^]^ The *I*–*T* response of heterojunction device under a series of X‐ray dose rates (75.2 µGy·s^−1,^ 238.6 µGy·s^−1^, 402.0 µGy·s^−1^ and 565.4 µGy·s^−1^) is shown in Figure 3g. As the X‐ray dose rate increases, the photocurrent of the device shows a rising trend. Specifically, at a dose rate of 75.2 µGy·s^−1^, the sensitivity reaches as high as 1885.1 µC Gy^−1^ cm^−2^ (Figure 3h). Furthermore, the self‐driven detection behavior of the device at zero bias voltage is studied. As shown in Figure S11 (Supporting Information), the Te/FAPbI₃ heterojunction device exhibits stable response behaviors under periodic light stimulation at zero bias voltage. The calculated on/off current ratio (I_photo_/I_dark_) of the device reaches 498. In addition, the spectral response of our heterojunction devices is compared to that of advanced reported detectors, as shown in Figure 3i (**Table** 2).^[^ ^24^, ^35^ ^]^ It is evident that our devices exhibit a broader spectral response range.

**Figure 4:**
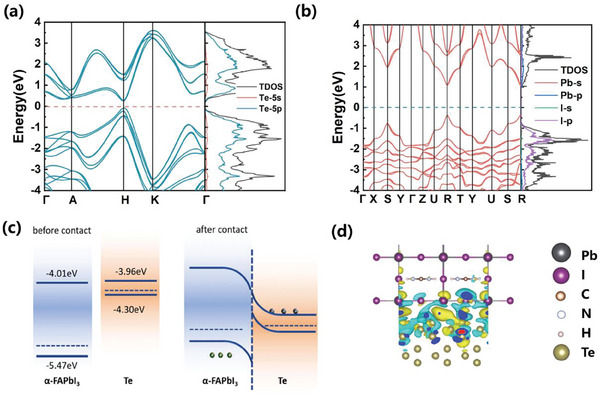
Band structure of a) hexagonal tellurium and b) trigonal FAPbI_3_ perovskite calculated by HSE hybrid functional. c) Te/FAPbI_3_ heterojunction band alignment before and after contact. d) Differential charge of Te/FAPbI_3_ heterojunction surface. The cyan (yellow) part shows a decrease (increase) in charge density.

To explore the underlying mechanism of high responsivity and self‐powered response of heterojunction photodetector, we exploited the first principle calculation to ascertain the band structure of Te^[^ ^35f^ ^]^ and FAPbI_3_,^[^ ^36^ ^]^ which are plotted in **Figure** **4**a,b. The band gap of hexagonal tellurium is assessed to be 0.34 eV.^[^ ^37^ ^]^ In the meanwhile, perovskite has a relatively wide band gap (1.45 eV), which is consistent with the experimental optical band gap. The positions of valence bands and Fermi levels for Te and FAPbI_3_ (Figures S13 and S14, Supporting Information) were determined by Ultraviolet Photoelectron Spectroscopy. The valence band maximum and conduction band minimum of tellurium are both higher than those of FAPbI_3_, forming a type‐II band alignment.^[^ ^38^ ^]^ As shown in Figure 4c, the formation process of built‐in electric field can be understood as follows. Initially, owing to the higher Fermi level of Te, the electrons in Te are transferred to the FAPbI_3_ while holes of FAPbI_3_ migrate to Te side until reaching equilibrium.^[^ ^13^ ^]^ As a result, a space charge region forms at the Te and perovskite interface, and a built‐in electric field is established, with the direction of the electric field pointing from Te toward FAPbI_3_.^[^ ^24^ ^]^ When exposed to high energy photons in the UV and Vis wavelength regions, both Te and perovskite form photo‐generated electrons and holes. Under zero or negative bias on the perovskite side, the external and built‐in electric fields align. This alignment causes photo‐generated electrons to move from the conduction band of perovskite to the conduction band of Te, resulting in photocurrent generation. Under positive bias on the perovskite side, the external field opposes the built‐in field. When the external field exceeds the built‐in field, photo‐generated holes move from the perovskite valence band to Te for negative electrode collection, while photo‐generated electrons transfer from the Te conduction band to the perovskite conduction band for collection.^[^ ^38^ ^]^ Conversely, when the incident photon energy is greater than the bandgap of Te but less than that of the perovskite (such as NIR wavelength region), photo‐generated electrons and holes form only in the Te layer. Under positive bias on the perovskite side, photo‐generated electrons transfer from the Te conduction band to the perovskite conduction band, thereby generating photocurrent. It is noted that the pure perovskite device without Te layer is incapable of effectively detecting photons in the near‐infrared (NIR) region, as such photons with energies lower than the perovskite bandgap are insufficient to induce photo‐generated electron–hole pairs within the device.^[^ ^24^ ^]^ Thus, the combination of Te/perovskite heterojunction help to broaden the range of light wavelength response compared with pure perovskite. Figure 4d further illustrates the charge transfer at the heterojunction interface, revealing effectively charge transfer between the two intimately contacted materials, where the cyan and yellow regions represent charge depletion and accumulation, respectively.

**Figure 5:**
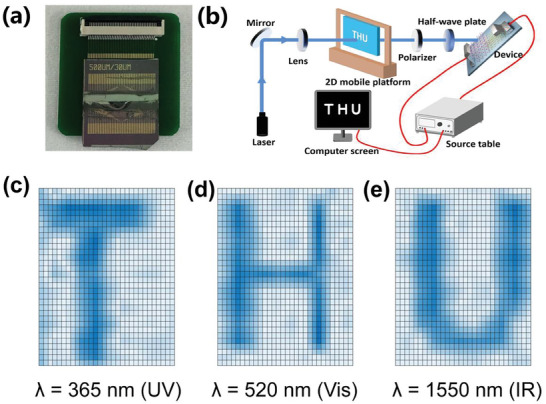
a) The optical photo of 40 × 1 line array Te/FAPbI_3_ heterojunction device. b) schematic diagram of imaging measurement system. c) Imaging result of letters “THU” under illumination at different wavelength of 365, 532, and 1550 nm under the voltage bias of 1 V.

The design of our heterojunction device facilitates broadband imaging, as depicted in **Figure** **5**a. A proof‐of‐concept linear array has been constructed to illustrate the scalability of heterojunction device pixels for integration.^[^ ^39^ ^]^ Importantly, heterojunction device enables the reconstruction of broadband images in various lighting conditions, including ultraviolet (365 nm with 5.0 mW cm^−2^), visible light (532 nm with 10.0 mW cm^−2^) near‐infrared (1.55 µm with 10.0 mW cm^−2^), utilizing a single‐pixel scanning approach (Figure 5b). The imaging results of the “THU” characters are illustrated in Figure 5c–e under a voltage bias of 1 V. The letters captured in the image have clear outlines, demonstrating the capacity of the heterojunction device for wide‐spectrum imaging.

## 3 Conclusion

In conclusion, we demonstrate a sensor capable of detecting from X‐ray to near‐infrared (NIR) on a single device using a Te transmission layer design, which simultaneously reduces dark current noise and broadens the long‐wavelength response spectrum. First‐principles calculations were used to select tellurium (Te) as a charge‐blocking layer in the perovskite, achieving a significant offset that effectively reduces the device dark current level and suppressed the noise current power. The low‐dark current noise facilities an X‐ray sensitivity reaching 1885.1 µC Gy^−1^ cm^−2^. The charge‐blocking layer also possesses a narrow bandgap, enabling the detection of long‐wavelength radiation, with a detectivity at 1550 nm reaching 6.8 × 10^9^ Jones. The band interface features a type‐II heterojunction structure, which facilitates efficient detection under self‐driven conditions. Furthermore, a 40 × 1 scale sensor array is fabricated to demonstrate the scalability. This work is expected to advance the application of perovskite‐based wide‐spectrum devices.

**Table 1:** Fitting parameters of time‐resolved photoluminescence for FAPbI_3_ and FAPbI_3_/Te utilizing a bi‐exponential decay function.

Materials	A_1_	A_2_	τ_1_ [ns]	τ_2_ [ns]	τ_ave_ [ns]
FAPbI_3_	0.43	0.37	10.84	253.18	241.79
FAPbI_3_/Te	0.64	0.35	3.71	14.07	10.79

**Table 2:** Benchmark of wavelength detection range of broadband spectrum photodetector.

Device	Wavelength	R or Sensivity	D*[Jones]	On/off time	References
Perovskite/Te	X‐ray‐1550nm	0.34 A/W	1 × 10^10^	10/25.66ms	This work
PbS:P3HT:PCBM:ZnO	350–930 nm	1.24 A/W	2.26 × 10^11^	160/80µs	[35h]
CH_3_NH_3_PbI_3_/ITO	365–780 nm	3.49 A/W	‐	0.9/1.7s	[35g]
Perovskite/polymer hybrid	400–700 nm	0.3 A/W	3 × 10^13^	4.82/4.3 µs	[35f]
InSe/ReS_2_	365–965 nm	1921 A/W	6.51 × 10^13^	21.6/43.2 ms	[35e]
GaSb	405–1550 nm	1.6 × 10^3^ A/W	5.7 × 10^9^	4.5/200 ms	[35d]
CH_2_NH_3_PbI_3_/TiO_2_/Si	400–1100 nm	62.5 A/W	4.85 × 10^13^	0.89/0.42 s	[35c]
PbS /ZnO	365–970 nm	14.8 A/W	3.78 × 10^10^	‐	[24]
β‑Ga_2_O_3_/Au/MAPbBr_3_	240–520 nm	0.47 A/W	7.3 × 10^10^	6.1/334 µs	[41]
Perovskite/Germanium	405–980 nm	228 A/W	1.6 × 10^10^	1.8/5.1 ms	[35b]
InSe	450–785 nm	12.3 A/W	5.47 × 10^10^	40‐50/50‐60 ms	[42]
In_2_Te_3_	350–1090 nm	0.3 A/W	‐	0.07/0.07 s	[43]
MAPbBr_3_ single‐crystal	X‐ray	80 µC Gy_air_^−1^ cm^−2^	6.6 × 10^11^	216/200µs	[35a]
CsPbBr_3_	X‐ray	0.172 A/W	4.8 × 10^12^	0.14/0.12 ms	[44]
MAPbI_3_	X‐ray	1270 µC mGy_air_^−1^ cm^−3^	2.43 × 10^12^	0.3/0.31 µs	[45]

## 4 Experimental Section

### Fabrication of Perovskite Film

0.1720 g FAI and 0.4610 g PbI_2_ powder were dissolved in the 750 mL mixed dimethyl sulfoxide (DMSO) and dimethylformamide (DMF) solution (4:1 molar ratio) as precursors. The precursors solution was packed in a sealed bottle and magnetically stirred at 70 °C for an hour until the powder was completely dissolved. By the way, the anti‐solvent was composed of hexyl hydride and ethyl acetate (7:3 volume ratio). In this experiment, the perovskite film was prepared by two‐step spin‐coating. The first step was to lay 50 µL of perovskite precursor solution on the silicon substrate surface and subsequently rotated it for 10s at 1000 rpm. The next step was to drop 170 µL of anti‐solvent at the moment when the rotation speed changed to 3000 rpm and then continued to spin for 30 s. After that, the perovskite was annealed at 180 °C for 15min.^[^ ^40^ ^]^

### Growth of Tellurium Film

Te films were deposited by high vacuum thermal evaporation coating equipment with Te pellets. When the base pressure in the chamber reached 4.2 × 10^−5^ Pa, the inverter evaporation power supply voltage was adjusted to 1.2 V and the corresponding current was set to 18 A. During the evaporation, the deposition rate was controlled approximately at 1.2 Å s^−1^. The thickness of Te films was regulated ≈50 nm. After evaporation, the samples could be taken out until they came down to room temperature.

### Fabrication of Heterojunction Photodetector

The fabrication process of the heterojunction is shown in Figure S1 (Supporting Information). 1) First, photolithography was performed on a 500 µm thick silicon wafer with a 500 nm thermal oxide layer. Positive photoresist AZ601 was used, and the Ag electrode pattern was defined by the lift‐off method. A 50 nm thick Ag electrode was deposited via magnetron sputtering method. 2) Polyimide tape was used as a hard mask to protect one side of the electrode, partly exposing the channel region between the electrodes. A 50 nm thick Te layer was then deposited using thermal evaporation method, and the polyimide tape hard mask was removed. 3) The polyimide tape hard mask was again used to protect the other side of the electrode. The perovskite film was coated using a two‐step spinning process. 1.33 mmol L^−1^ FAPbI_3_ DMSO/DMF solution serves as the precursor, using mix hexane/ethyl acetate as the anti‐solvent. After deposition, the polyimide tape was removed, resulting in an Ag‐perovskite‐Te‐Ag lateral heterojunction structure. The channel length and width of the photodetector were 1000 and 30 µm, respectively.

### Material Characterization

X‐ray diffraction (XRD) pattern was performed utilizing Rigaku Corporation instrument(D/max‐2550) with a 2θ from 10° to 60° in step of 0.1°. X‐ray photoelectron spectroscopy (XPS) measurement was conducted on Shimadzu Corporation (AXIS Supra). The surface potential and roughness of as‐prepared samples was characterized by atomic force microscope (AFM, Bruker Dimension ICON). The morphologies and elemental chemical compositions of samples were detected by scanning electron microscope (SEM) with EDX. The data of time‐resolved photoluminescence was analyzed by Edinburgh FLS920. Raman spectrum was using HORIBA Corporation (HR800) under 532 nm laser excitation.

### X‐Ray Test Configuration

In this experiment, a tungsten anode X‐ray tube capable of producing Bremsstrahlung radiation with a maximum energy of 100 keV was employed. The dose rate calibration relied on a reference ionization chamber (models: PTW‐32002 and NIM‐MEFAC‐01, National Institute of Metrology, Beijing, China). For evaluating the electrical properties of the devices, a semiconductor characterization system (Keithley 2636B) was utilized.

### Computational Details

The calculation method in this work was based on the first‐principles, using the code in the VASP software package based on the density functional theory (DFT). The Perdew‐Burke‐Ernzerhof (PBE) exchange correlation function was used to describe electron interactions in the generalized gradient approximation (GGA). According to the convergence test, in the process of calculating the structure optimization, electron self‐consistency and band structure, the energy cutoff was set to 500 eV. The precision of energy convergence and the force convergence standard was 1 × 10^−6^ eV atom^−1^ and 0.01 eV Å^−1^. The thickness of the vacuum layer was set as 15 Å. 8 × 8 × 8 k‐point grid based on Monkhorst‐Pack was applied to sample the Brillouin zone and density of states (DOS).

### Statistical Analysis of TRPL data

Bi‐exponential decay function of TRPL:

(3) $$I=A_{1}\exp\left(-\frac{t-t_{0}}{\tau_{1}}\right)+A_{2}\exp\left(\frac{t-t_{0}}{\tau_{2}}\right)$$

where t_0_ represents the beginning time of decay process, A_1_ and A_2_ were attenuation amplitude of each channel, respectively. τ_1_ and τ_2_ were corresponding time of decay process.

The average lifetime was derived from the following equation

(4) $$\;\tau_{ave}=\frac{A_{1}\tau_{1}^{2}+A_{2}\tau_{2}^{2}}{A_{1}\tau_{1}+A_{2}\tau_{2}}$$

## Conflict of Interest

The authors declare no conflict of interest.

## Author Contributions

Y.‐C.H. and G.‐H.D. contributed equally to this work. T.R. supervised the project. G.D., D.X., H.T., Y.Y., and T.R. conceived and designed the experiments. Y. H., G.D. performed the experiments. Y.H. contributed to the first‐principles calculation and recognition simulation. Y.H. and G.D. contributed to the paper writing. All authors discussed and reviewed the manuscript.

## Supporting information



Supporting Information

## Data Availability

The data that support the findings of this study are available from the corresponding author upon reasonable request.
