# Supplementary material for: Band Tailoring Enabled Perovskite Devices for X‐Ray to Near‐Infrared Photodetection
Source: Adv Sci (Weinh). 2025 Jan 14;12(9):2414259. doi: 10.1002/advs.202414259 (PMC11884567; doi:10.1002/advs.202414259)
Supplement: Supplementary file 1 — Supporting Information [file ADVS-12-2414259-s001.pdf]

ADVANCED SCIENCE
Open Access

Supporting Information

for *Adv. Sci.*, DOI 10.1002/advs.202414259

Band Tailoring Enabled Perovskite Devices for X-Ray to Near-Infrared Photodetection

Yi-Chu He, Guan-Hua Dun, Jun Deng, Jia-Li Peng, Ken Qin, Jia-He Zhang, Xiang-Shun Geng, Min-Shu Zhang, Ze-Shu Wang, Yan Xie*, Zhao-Qiang Bai*, Dan Xie*, He Tian*, Yi Yang* and Tian-Ling Ren**

Supporting Figures

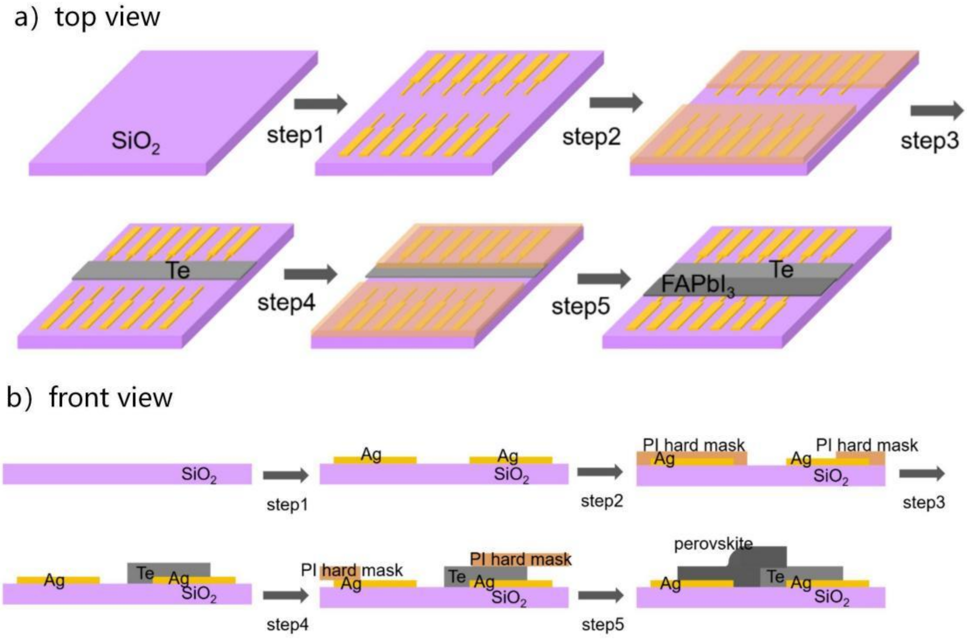

Figure S1 a) Overhead view and b) cross-view of the fabrication process of heterojunction photodetector.

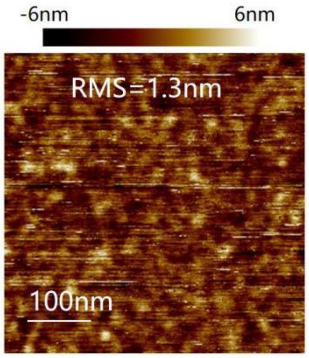

Figure S2 Surface roughness characterized by AFM.

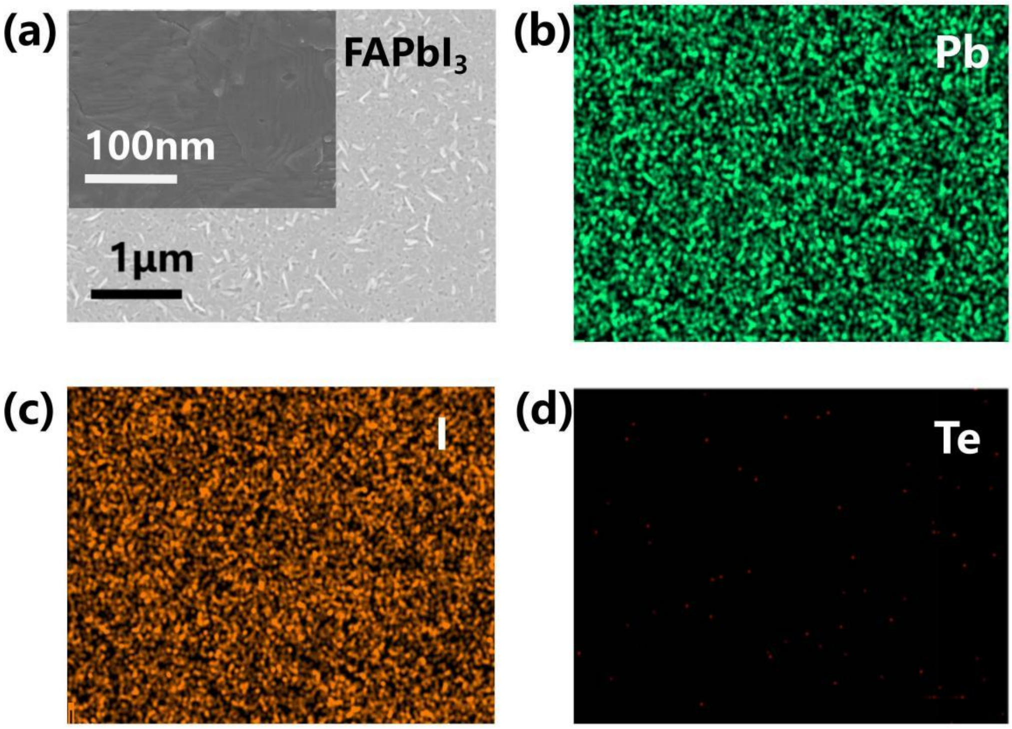

Figure S3 (a) SEM top-view image of $FAPbI_3$ perovskite film and (b-d) EDS mapping image of $FAPbI_3$ perovskite film on Te with Pb, I, Te elements.

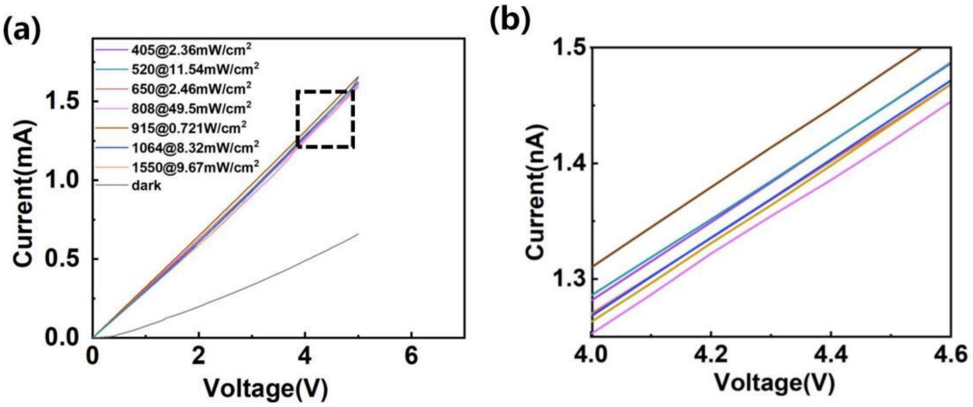

Figure S4 (a) I-V curves of Te sensor under variable wavelengths from 405 nm to 1550 nm. (b) The right is a magnified view of a specific section under bias of 4.0 V to 4.6 V.

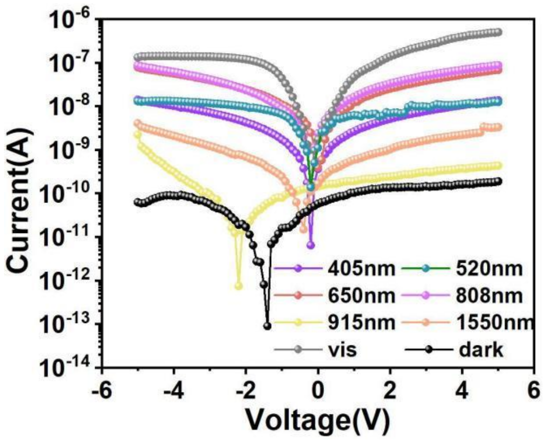

Figure S5 I-V curves of perovskite device under different illumination wavelength, including 405 nm, 520 nm,650 nm, 808 nm, 915 nm, 1550 nm, visible light and dark condition (Bias: 5 V).

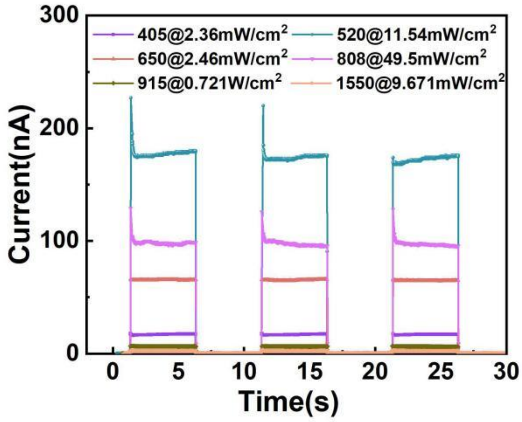

Figure S6 Dynamic photoresponse curves of $FAPbI_3$ photodetector under periodic illumination with wavelength range from 405 nm to 1550 nm at 5 V bias.

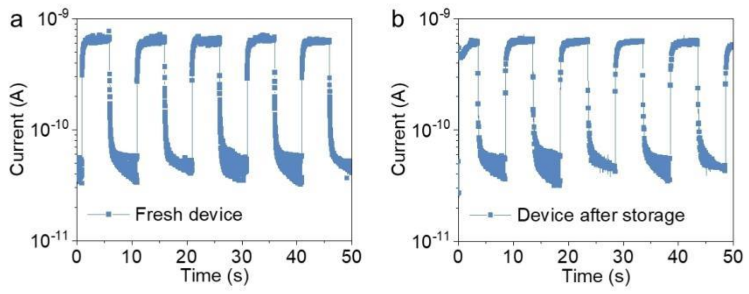

Figure S7 The photoresponse of $FAPbI_3$/Te fresh device and device after storage under ambient conditions for 5 months.

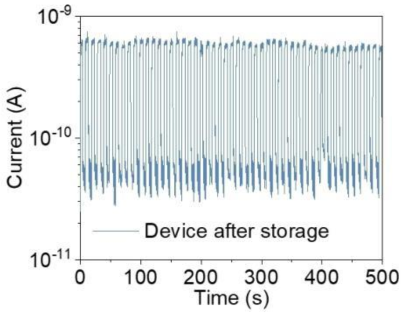

Figure S8 The photoresponse of $FAPbI_3$/Te device after storage under ambient conditions for 5 months in 50 light I_{on}/I_{off} illumination cycles.

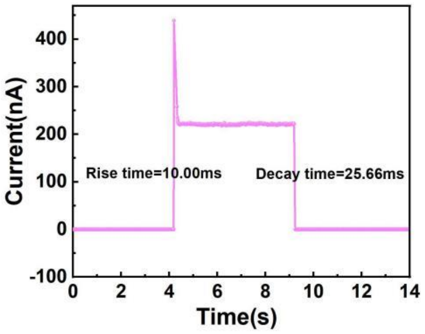

Figure S9 One cycle on/off switch photoresponse of Te/$FAPbI_3$ photodetector under irradiation to wavelength of 808 nm with 5 V bias.

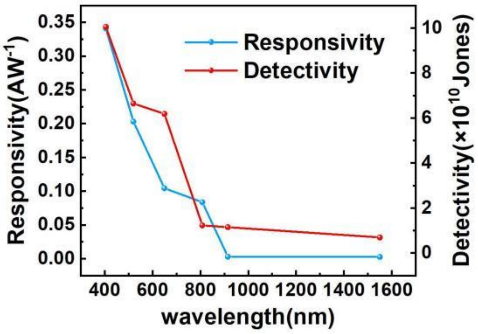

Figure S10 Responsivity and Detectivity of heterojunction with wavelength range from 405 nm to 1550 nm at 5 V bias.

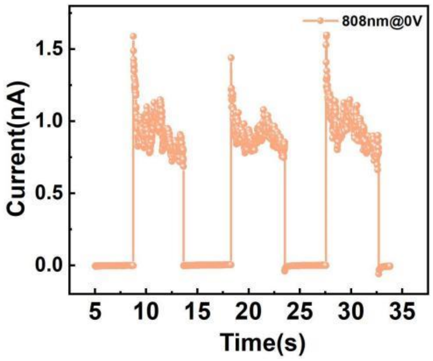

Figure S11 Three cycles on/off switch photoresponse of Te/$FAPbI_3$ device under irradiation to 808 nm with zero bias voltage.

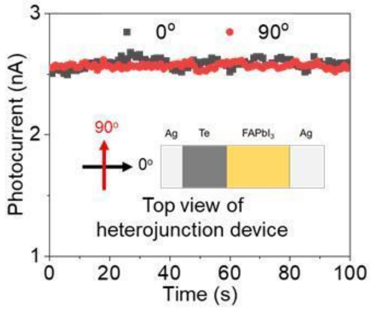

Figure S12 Polar diagram of the polarized photoresponsivity under 5 voltage bias for incident wavelength of 405 nm. And polarized light is set to parallel and perpendicular to the heterojunction device.

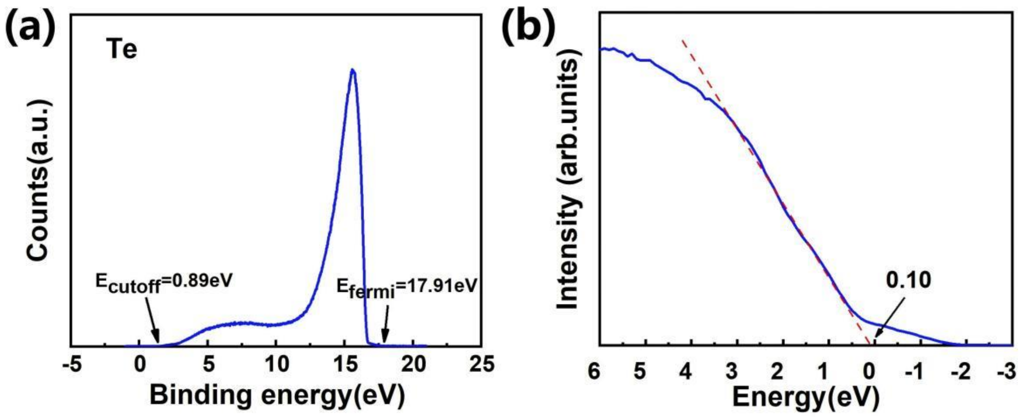

Figure S13 Energy spectrum characterization of Te films (a) UPS (b) XPS

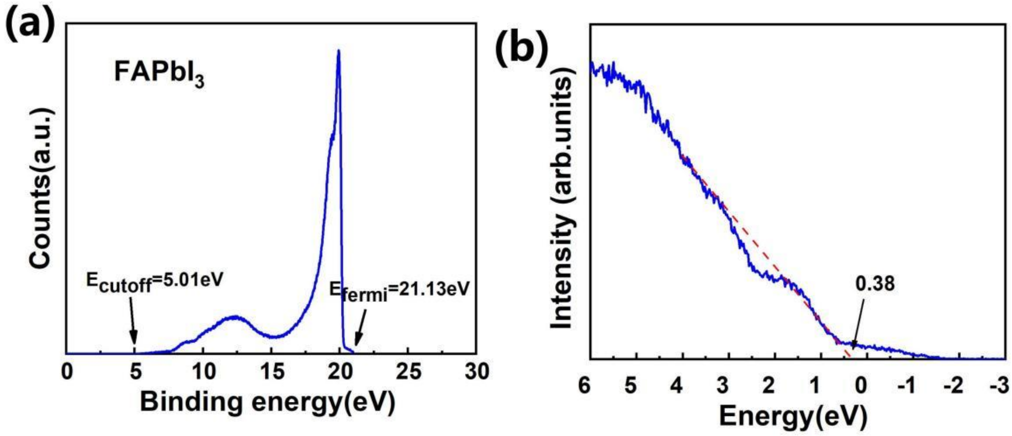

Figure S14 Energy spectrum characterization of $FAPbI_3$ (a) UPS (b) XPS

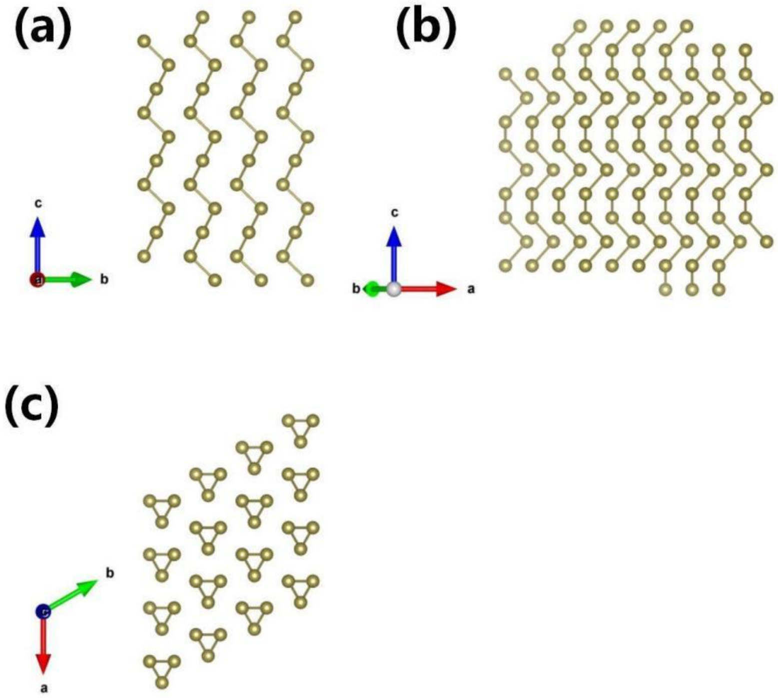

Figure S15 a) Front view, (b) side view and (c) top view of Te model

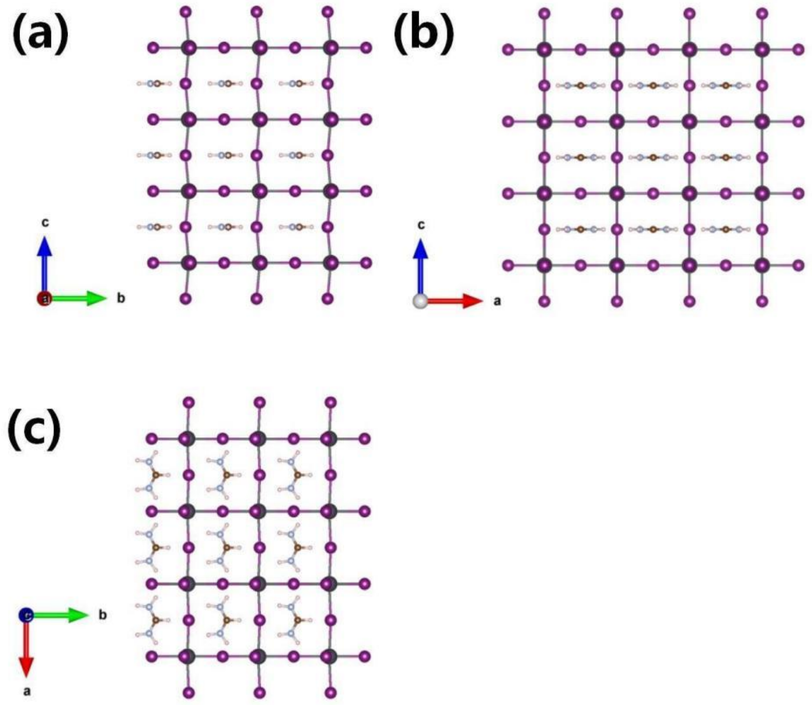

Figure S16 a) Front view, (b) side view and (c) top view of $FAPbI_3$ model

Note1： Distinguishment of material responsible for detection across different wavelength range

In the ultraviolet to visible range (400–900 nm), perovskite exhibits stronger absorption, which is consistent with its relatively wide bandgap (1.46 eV). In this region, the absorption of perovskite is larger than Te, and therefore the perovskite plays the primary role in detection. In the infrared range (1200 nm–1700 nm), the perovskite shows a low absorption, which in because that low-energy photons in this infrared range are insufficient to excite band-band transitions in perovskite. In contrast, Te exhibits significantly stronger absorption in the infrared range, which is consistent with its narrow bandgap (0.34 eV). Thus, detection in this region is primarily attributed to Te. In the range of 900–1200 nm, the absorption capacities of perovskite and Te are comparable, and the absorption in this region can be attributed to a combined response from both perovskite and Te.
